# Exploring the complexities of vaccine sentiment among healthcare and public health professionals: essential strategies for encouraging vaccine uptake

**DOI:** 10.3389/fpubh.2025.1537255

**Published:** 2025-05-09

**Authors:** Mariana Gonzalez Utrilla, Sarah Yingli Tan, Michael Moore, Marta Lomazzi

**Affiliations:** ^1^World Federation of Public Health Associations, Geneva, Switzerland; ^2^Ministry of Health Holdings, Elementum, Singapore, Singapore; ^3^Institute of Global Health, University of Geneva, Geneva, Switzerland

**Keywords:** vaccines, policy, healthcare workers, health, prevention

## 1 Introduction

Healthcare workers (HCWs) are at a significantly higher risk of contracting infectious diseases due to their increased exposure. In low- and lower-middle-income countries, the vaccination of HCWs is particularly crucial yet faces numerous implementation challenges. In a review of COVID-19 vaccination hesitancy in HCW worldwide, it was noted that vaccine hesitancy is a documented and persistent phenomenon, with varying prevalence globally and significant implications for public trust and vaccine uptake (1).

Emphasizing the importance of this issue, the WFPHA policy statement “Understanding Vaccine Sentiment: Identifying Obstacles and Opportunities to Boost Vaccination Uptake Among Healthcare and Public Health Professionals” highlights the need for robust policies to ensure the routine vaccination of HCWs. This policy statement serves as a vital instrument for influencing policymakers and healthcare organizations to prioritize and enhance vaccination efforts among healthcare workers globally.

## 2 Exploring the complexities of vaccine sentiment among healthcare and public health professionals is essential for formulating effective strategies aimed at encouraging vaccine uptake

### 2.1 Vaccine efficacy, sentiment, and barriers to uptake

The landscape of public health is marked by the ongoing struggle to combat communicable diseases, with vaccination serving as a cornerstone of preventive healthcare strategies. Vaccines have historically played a pivotal role in reducing the spread of infectious diseases, contributing significantly to improvements in global health outcomes. Consider the cases of smallpox and the veterinary disease rinderpest, both of which were successfully eradicated. Furthermore, vaccinations have significantly improved the control of diseases such as polio and measles (2). Despite the undeniable efficacy of vaccines, the persistent challenge of vaccine hesitancy among healthcare professionals poses a significant barrier to achieving widespread immunization coverage. The importance of dealing with these barriers is emphasized by the World Health Organization classifying vaccine hesitancy as one of the top 10 global health threats (3).

At the heart of public health efforts lies the imperative to promote trust and confidence in vaccination programs. Healthcare professionals, as frontline advocates of public health, wield considerable influence in shaping public perceptions and behaviors regarding vaccination. Their endorsement of vaccination not only bolsters individual and community immunity but also serves as a catalyst for broader societal acceptance of immunization efforts through patient interactions and broader community-level engagement efforts (4).

However, amidst the triumphs of vaccination, there exists a complex intersection of attitudes and beliefs that underpin vaccine sentiment among healthcare professionals. Vaccine hesitancy, characterized by doubts, concerns, or refusal of vaccination despite its availability, poses a multifaceted challenge that demands understanding and targeted interventions.

The roots of vaccine hesitancy among healthcare professionals intertwine with historical, cultural, and socio-economic factors, alongside contemporary influences such as misinformation, mistrust in governments, and hesitancy toward medical interventions (5). The ramifications of vaccine hesitancy resonate across healthcare systems, undermining efforts to mitigate the burden of vaccine-preventable diseases and safeguard public health. One such example is the COVID-19 pandemic, which highlighted the detrimental impacts of misinformation and systemic distrust on public health initiatives, resulting in vaccine hesitancy amongst healthcare workers and the public (6).

Understanding the intricacies of vaccine sentiment among healthcare professionals is not merely an academic pursuit but a pragmatic necessity in devising tailored strategies to enhance vaccine acceptance. Recognizing the diverse motivations and barriers that shape individual attitudes toward vaccination is pivotal in fostering dialogue, dispelling misconceptions, and cultivating a culture of evidence-based decision-making within healthcare settings.

The imperative to address vaccine hesitancy among healthcare professionals extends beyond individual clinical encounters to encompass systemic reforms and collaborative initiatives that prioritize the promotion of vaccination as a public health imperative. Embracing a holistic approach that integrates education, advocacy, and community engagement is essential in fostering a supportive environment conducive to vaccine acceptance and uptake. To enhance the efficacy of strategies and recommendations to promote vaccine acceptance, it is essential to integrate culturally appropriate practices and to consider the diversity of healthcare environments (7).

In navigating the complexities of vaccine hesitancy among healthcare professionals, it is necessary for public health stakeholders to redouble their efforts in championing the importance of vaccination as a cornerstone of preventive healthcare. By fostering trust, promoting transparency, and engaging in meaningful dialogue, barriers to vaccine acceptance can be surmounted and chart a course toward a future where immunization is embraced as a fundamental pillar of global health security.

### 2.2 Recommendations from the world federation of public health associations policy

With these in mind, the World Federation of Public Health Associations (WFPHA)'s International Immunization Policy Task Force undertook a series of focus group meetings with national public health associations and vaccination experts. They elaborated on the key pillars to prevent vaccine hesitancy and increase acceptance of vaccines within the healthcare workforce. The main recommendations that were discussed formed the basis of the official WFPHA policy “*Understanding Vaccine Sentiment: Identifying Obstacles and Opportunities to Boost Vaccination Uptake Among Healthcare and Public Health Professionals”* (Table 1) (4).

**Table 1:** Fiver pillars of preventing vaccine hesitancy.

**Pillar of action**	**Recommendations**	
Understand and empower	- Actively listen to health workforce concerns. - Conduct advocacy campaigns across Public Health Associations, authorities, and NGOs. - Coordinate efforts across key agencies and sectors. - Address concerns and underlying factors. - Encourage proactive engagement of healthcare workers.	
Communicate effectively	Share positive and trusted messages	- Maintain a positive, encouraging tone in promoting vaccination. - Disseminate data highlighting benefits of vaccination. - Refresh and reinforce positive messages. - Use visually compelling tools like infographics.
	Risk assessment communication campaign	- Transparently share data on adverse events following immunization. - Emphasize distinction between true vaccine-related side effects and stress-induced responses. - Design awareness campaigns to restore trust and encourage transparent reporting. - Emphasize the low risk of vaccination compared to the risk of the disease.
	Designate specific public health vaccination advisors or communicators	- Appoint advisors to provide tailored, evidence-based responses to health professionals. - Address concerns without judgment. - Possess understanding of cultural and other relevant factors.
Enhanced accessibility	- Ensure easy access to vaccinations by eliminating local and temporal barriers. - Offer vaccinations at easily accessible public spaces. - Reduce complexity in delivery of vaccinations. - Encourage vaccination through pharmacy. - Provide vaccinations without cost. - Use inclusive language in communication materials. - Offer vaccinations to multiple generations simultaneously. - Encourage receiving recommended vaccinations together for comprehensive protection. - Advocate for combined flu and COVID-19 vaccination options.	
Continuous education and training	- Integrate vaccination training into professional programmes. - Ensure ongoing training for healthcare workers. - Provide education on community immunity and vaccine benefits. - Empower healthcare workers to confidently communicate vaccination importance. - Offer specific training addressing doubts, questions, and barriers to vaccine acceptance.	
Make community leaders allies	- Collaborate with community leaders to amplify positive vaccination messages. - Utilize insights of community leaders into cultures and belief systems. - Engage leaders to dispel doubts and spread positive messages about vaccination. - Strengthen trust and cooperation through collaborative efforts with community leaders.	

The pillars proposed by the WFPHA have a rationale that effectively aim to counteract vaccine hesitancy and increase vaccination uptake within the healthcare workforce. The five key pillars are namely—Understand and Empower, Communicate Effectively, Enhance Accessibility, Continuous Education and Training, Make Community Leaders Allies.

#### 2.2.1 Pillar 1—understand and empower

The first step in counteracting vaccine hesitancy is to understand the underlying causes of these concerns and to formulate strategies that counter these apprehensions. Establishing strong working relationships with Public Health Associations (PHAs), authorities, non-governmental organizations (NGOs) and other related agencies are key to organizing campaigns.

These initiatives create open dialogue where healthcare professionals can voice their concerns and have them addressed. This fosters a sense of empowerment and engagement, allowing them to come together, share their worries, and find reassurance and support. Empowering healthcare professionals to take ownership. Appropriate initiatives can cultivate a culture of accountability and responsiveness to drive positive change. This sense of empowerment ensures that healthcare professionals' voices are heard, which in turn can significantly improve vaccine uptake by addressing their concerns and fostering a more supportive environment.

#### 2.2.2 Pillar 2—communicate effectively

In counteracting misinformation and building confidence in immunization, it is imperative to disseminate evidence-based benefits of vaccination in a positive and encouraging manner and to share this information in visually captivating and easily digestible formats such as infographics. Information sheets need to be provided in a culturally and socio-economically sensitive manner at no cost. In this digital era where perceptions are easily shaped by encounters on social media and digital platforms, it is also crucial to integrate digital strategies to improve health literacy and dispel myths in healthcare workers and the wider community.

Evidence has shown that integrating interactive digital methods can significantly enhance health literacy within communities. For instance, a study on the World Health Organization's (WHO) “Go Viral!” online game demonstrated several positive outcomes: it increased the perceived deceptiveness of COVID-19 misinformation, boosted confidence in recognizing and debunking false information, and reduced the inclination to share such misinformation (8). As part of a comprehensive promotional campaign, it is also essential to ensure transparency in sharing statistics on Adverse Events Following Immunization (AEFI) to prevent misinformation. AEFIs should be thoroughly investigated and reported. When an event is found to be coincidental rather than caused by the vaccine, it should be explicitly communicated to the public to prevent misconceptions. For instance, stress responses can coincide with vaccination but are not true side effects. Public information should clarify this distinction. Additionally, programs to manage stress responses, such as the CARD system (Comfort, Ask, Relax, and Distract), should be readily available to support individuals during vaccination (9). The risk associated with AEFIs ought to be put in context against the risk associated with the disease.

Communicating vaccination benefits while transparently addressing true side effects and distinguishing them from stress-induced responses fosters trust in government recommendations and dispels myths. To ensure all the aforementioned are evident in each vaccination campaign, a designated public health vaccination advisor should oversee all campaign communications, ensuring they also take into consideration socio-economic, cultural and religious factors to effectively reach healthcare workers and the wider population.

#### 2.2.3 Pillar 3—enhanced accessibility

Enhancing accessibility to vaccinations is fundamental for overcoming hesitancy among healthcare workers. This approach should not only focus on the ease of obtaining vaccinations but also consider accessibility in a comprehensive, holistic manner. Healthcare facilities should prioritize making the vaccination process as easy and convenient as possible for their staff, thereby removing barriers and encouraging higher vaccination rates. This may involve offering vaccination during work hours, onsite vaccination clinics, and flexible scheduling options. Additionally, addressing any logistical challenges, such as childcare or transportation barriers, can help to improve vaccination rates. To improve vaccination accessibility, vaccines should ideally be readily available at all times for all age groups. Encouraging health professionals to receive all recommended vaccinations in a single visit is a strategic approach to promoting immunization and protecting healthcare workers against various preventable diseases. Evidence suggests that co-administering vaccines can significantly increase their uptake (10, 11). Additionally, healthcare workers should have access to the necessary vaccinations free of charge. Access through pharmacy and the use of appropriately trained pharmacists (as healthcare professionals themselves) may also increase access. In Italy, pharmacy-based vaccinations were introduced to the vaccination programme, making them suitable support structures for vaccination campaigns targeting the adult, older adults and vulnerable populations.

Strategies are in line with the WHO's Immunization Agenda 2030 and a systemic review highlighting that the above measures have been proven to increase vaccination in healthcare workers (10).

#### 2.2.4 Pillar 4—continuous education and training

Building trust is essential for addressing vaccine hesitancy. Limited knowledge and training in vaccinations appears to be a factor in low immunization rates in healthcare workers. Healthcare facilities should create open and transparent communication channels to ensure that healthcare workers feel comfortable raising questions and voicing concerns. As part of a comprehensive national approach, formal training programmes are needed to educate healthcare workers, address their questions and doubts and to alleviate barriers to vaccine acceptance. This may involve establishing or enhancing vaccine advisory committees. Keeping healthcare workers abreast of the latest vaccine-related information is key to ensuring increased vaccine uptake and concurrently empowering them to be confident frontline advocates of immunization (11, 12).

#### 2.2.5 Pillar 5—make community leaders allies

Collaborative efforts with community leaders can be an impactful approach to improving vaccine rates in healthcare workers and the community. Indeed, healthcare workers, being members of their communities, can be influenced by the actions and opinions of community leaders. Community leaders ranging from religious leaders to influencers guide the public opinion and can have a huge effect on vaccine uptake rates (12). They may influence not only the general public but also healthcare professionals, especially in contexts where these leaders hold moral or social authority. Their endorsement of vaccination can reinforce professional confidence, address value-based concerns, and create a shared sense of responsibility that bridges personal belief systems with public health goals. Religious leaders in India for instance, are involved in vaccine campaigns to counter misinformation pertaining to infectious diseases. They serve as influential stakeholders in a prime position to shape perspectives and improve public confidence in vaccine campaigns. One such example is the CIVIC Project in Mewat, India which demonstrated that involving religious faith leaders provided reassurance to the community and created a platform for two-way dialogue and dispelling misconceptions thus increasing vaccine confidence (13). By harnessing the influence of community leaders, positive messages about vaccinations can be promoted, creating a favorable domino effect where myths are debunked, and vaccine uptake rates increase in both healthcare workers and the public.

### 2.3 A comprehensive approach to encouraging vaccination uptake

A wide array of factors influences vaccine sentiment in healthcare and public health professionals, and it is imperative to explore their intricacies in order to formulate effective strategies to encourage vaccine uptake. This is especially important in low and middle-income countries, where healthcare infrastructure is often limited or largely inaccessible. Overcoming vaccine hesitancy is vital for achieving effective immunization coverage and ensuring the success of preventive health programs, as the screening and care systems in these regions may be weak. The WFPHA's policy recommendations must be implemented cohesively rather than in isolation, integrating all five pillars to build a holistic strategy that promotes widespread vaccination uptake. For instance, Italy's COVID-19 vaccination campaign exemplified effective integration between national coordination and regional implementation, whereby centralized guidelines were operationalized through region-specific delivery models. Several regions deployed mobile vaccination units to reach remote populations, utilized pharmacies in urban settings to enhance accessibility, and established temporary clinics within community hubs to address local needs, all within the framework of the national strategy. By implementing these five key pillars, healthcare facilities can create a culture of vaccine confidence and empower healthcare workers to make informed decisions about vaccination. This will ultimately contribute to a safer work environment for healthcare workers and better protect the patients they serve.
